# Phylogeny of the Genus *Chrysanthemum* L.: Evidence from Single-Copy Nuclear Gene and Chloroplast DNA Sequences

**DOI:** 10.1371/journal.pone.0048970

**Published:** 2012-11-01

**Authors:** Ping-Li Liu, Qian Wan, Yan-Ping Guo, Ji Yang, Guang-Yuan Rao

**Affiliations:** 1 College of Life Sciences, Peking University, Beijing, China; 2 College of Life Sciences, Beijing Normal University, Beijing, China; 3 School of Life Sciences, Fudan University, Shanghai, China; CNR, Italy

## Abstract

*Chrysanthemum* L. (Asteraceae-Anthemideae) is a genus with rapid speciation. It comprises about 40 species, most of which are distributed in East Asia. Many of these are narrowly distributed and habitat-specific. Considerable variations in morphology and ploidy are found in this genus. Some species have been the subjects of many studies, but the relationships between *Chrysanthemum* and its allies and the phylogeny of this genus remain poorly understood. In the present study, 32 species/varieties from *Chrysanthemum* and 11 from the allied genera were analyzed using DNA sequences of the single-copy nuclear *CDS* gene and seven cpDNA loci (*psbA-trnH, trnC-ycf6, ycf6-psbM, trnY-rpoB, rpS4-trnT, trnL-F*, and *rpL16*). The cpDNA and nuclear *CDS* gene trees both suggest that 1) *Chrysanthemum* is not a monophyletic taxon, and the affinity between *Chrysanthemum* and *Ajania* is so close that these two genera should be incorporated taxonomically; 2) *Phaeostigma* is more closely related to the *Chrysanthemum*+*Ajania* than other generic allies. According to pollen morphology and to the present cpDNA and *CDS* data, *Ajania purpurea* is a member of *Phaeostigma*. Species differentiation in *Chrysanthemum* appears to be correlated with geographic and environmental conditions. The Chinese *Chrysanthemum* species can be divided into two groups, the *C. zawadskii* group and the *C. indicum* group. The former is distributed in northern China and the latter in southern China. Many polyploid species, such as *C. argyrophyllum*, may have originated from allopolyploidization involving divergent progenitors. Considering all the evidence from present and previous studies, we conclude that geographic and ecological factors as well as hybridization and polyploidy play important roles in the divergence and speciation of the genus *Chrysanthemum*.

## Introduction

*Chrysanthemum* L. (Asteraceae-Anthemideae) is a genus associated with polyploidy and hybridization [1]–[5]. It is well known for its commercial chrysanthemum cultivars, which are globally important cut flowers and pot plants [6], [7]. This genus comprises about 40 species mainly distributed in East Asia, and the species diversity is centered in China [8]–[10]. The considerable variations in morphology and ploidy level are exhibited in this genus (from 2n = 2x = 18, to 2n = 36, 54, 72, up to 90) [1], [11]. Infraspecies and even infrapopulation variations in ploidy have been found in some cases [1], [2], [12]. In addition, many species of *Chrysanthemum* are narrowly distributed and habitat-specific [1], [4], [8], [9]. All this information indicates that speciation is associated with hybridization, polyploidy, and adaptation. So far, the evolutionary history of *Chrysanthemum* has not been well understood despite a lot of efforts [13], [14].

There is a great deal of controversy regarding its scientific name, entity and circumscription [15]–[17]. This situation has been improved through the resurrection of *Chrysanthemum* L. for the Asiatic species formerly under *Dendranthema* (DC.) Des Moul., and of *Glebionis* Cass. for the two Mediterranean species, *C. segetum* and *C. coronarium*, which had formerly been described under *Chrysanthemum* [8], [9], [18]. Because of the economic importance of garden chrysanthemums, an impressive amount of literature has accumulated on their breeding, cultivation, and origins [6], [7]. However, the relationships between the wild *Chrysanthemum* species remain poorly understood.

As shown in the morphology-based cladogram, *Chrysanthemum* has close relationships with *Brachanthemum, Arctanthemum, Tridactylina, Ajania*, and *Phaeostigma* [9]. It is distinguished from them by its obovoid, thin-walled, myxogenic cypselas without pappus, and involucral bracts with dark brown margins [9]. However, the relationships based on the morphological characters show little congruence with findings from molecular data [18]–[20]. In the molecular phylogeny of the subtribe Artemisiinae, there are two main groups, the *Artemisia-*group and the *Chrysanthemum-*group ( =  *Dendranthema* group) [19]. The latter, a strongly supported clade in the ITS tree, consists of *Chrysanthemum, Ajania, Arctanthemum*, and *Elachanthemum* [18], [19]. Moreover, the taxonomic positions of *Ajania* and *Phaeostigma* relative to *Chrysanthemum* have long been controversial due to the pollen exine ornamentations and other morphological traits, such as the type of ray floret [9], [21], [22]. *Ajania* and *Chrysanthemum* have the same, common type of pollen grains, Anthemis-type pollen with echinate, while the representative of *Phaeostigma* has Artemisia-type pollen with microechinate, measuring <1 μm in height [20]–[22]. *Ajania* and *Phaeostigma* shared the common disciform capitula, while *Chrysanthemum* has the radiate capitula [9]. Bremer and Humphries proposed that *Phaeostigma* might be a grade between *Ajania* and *Artemisia*, and suggested that circumscribing *Phaeostigma* requires more extensive study covering greater numbers of *Ajania* species [9]. *Ajania purpurea*, for example, is a small perennial only found in alpine habitats at elevations of 4800–5300 m, Xizang (Tibet) [8]. Based on our field observations, brownish style-branches and purple corolla with erect lobes distinguish it from other *Ajania* species. The phylogenetic position of this species needs to be clarified.

Interspecific relationships within *Chrysanthemum* are complicated because hybridization and polyploidy are common in this genus [1]–[5]. Although molecular phylogenetic data have been accumulated in the *Chrysanthemum* species, most of these data covered a small geographical range and focused primarily on garden cultivars and a few closely related wild species [23]–[25]. Two recent studies with larger numbers of samples and more molecular markers improved the phylogeny reconstruction of *Chrysanthemum* [13], [14]. However, the interspecific relationships still remain undetermined due to the limited informative sites of nuclear ribosomal and chloroplast DNA data. We here try to elucidate the evolutionary history of *Chrysanthemum* using DNA sequence data containing relatively large amounts of phylogenetic information from both nuclear and chloroplast genomes.

In terms of resolving the phylogeny of the taxa, particularly those with rapid speciation and complex relationships, single- or low-copy nuclear genes are superior to organelle and nuclear ribosomal DNA [26], [27]. First, they generally have high levels of variation to infer relevant gene trees with confidence. Second, they contain signals of hybridization and/or introgression if homeologous alleles are present in an individual or population. The analyses of single- or low-copy nuclear genes have helped with untangling the complex history of such taxa as *Cerastium, Paeonia, Oxalis*, and *Achillea* [28]–[31].

One candidate nuclear gene for this study is the chrysanthemyl diphosphate synthase gene (*CDS*). CDS catalyzes the condensation of two molecules of dimethylallyl diphosphate to chrysanthemyl diphosphate, an irregular monoterpene [32], [33]. Irregular monoterpenes are mainly found in plants of the tribe Anthemideae of Asteraceae [32]–[34]. *CDS* is a member of the farnesyl diphosphate synthase (FDS) gene family, but it differs from other members of the family by an N-terminal extension of 50 amino acids, which has been identified as a plastidial targeting peptide [32], [33]. Wang et al. isolated a fragment of *CDS* from *Chrysanthemum* × *morifolium* [35]. Their data were used to explore relationships among garden chrysanthemums and their affinities. However, that study provided little phylogenetic information due to the scarcity of samples from the wild species. Meanwhile, we have studied molecular evolution of the FDS gene family (Liu et al. unpublished). This allows us to identify *CDS* orthologs among the *Chrysanthemum* species and find variation-rich regions for the present study.

Based on a broad species sampling, we here conduct a phylogenetic study using DNA sequences of the nuclear single-copy *CDS* gene and seven cpDNA loci (*psbA-trnH, trnC-ycf6, ycf6-psbM, trnY-rpoB, rpS4-trnT, trnL-F*, and *rpL16* intron). Our major objectives are 1) to investigate the relationships between *Chrysanthemum* and its generic allies in the subtribe Artemisiinae, 2) to examine the interspecific relationships within *Chrysanthemum*, and 3) to verify polyploidy and hybridization speciation events common in *Chrysanthemum*.

## Materials and Methods

Forty-three species/varieties of *Chrysanthemum* and of seven other genera of the subtribe Artemisiinae (Anthemideae, Asteraceae) were sampled. Among them, 32 were from *Chrysanthemum*, four *Ajania*, two *Phaeostigma*, and one each from *Elachanthemum, Artemisia, Brachanthemum, Kaschgaria*, and *Stilpnolepis*, respectively (Table 1). For the diploid species and the narrowly distributed polyploid species, one individual from every studied population was sampled. For species with varying ploidy levels and a broad distribution, such as *C. indicum, C. naktongens*, and *C. zawadskii*, 2–5 populations were sampled with one individual from each population. For species with multiple varieties, one individual per variety studied was sampled. To incorporate information from garden chrysanthemums, two cultivated varieties of *C. morifolium* (accessions “ZY” and “HSZ”) representing two flower color series, yellow and white-purple, were included in this study (Table 1). Fresh leaves were collected in the field, desiccated, and stored in silica gel. The same plant individuals were sequenced at all the DNA loci. All the samples used in this study were collected by the senior author and his colleagues or cooperators, and no special permissions were required for collection. The locations are not privately owned or protected in any way and the field studies do not involve endangered or protected species. Voucher specimens were deposited in the Peking University herbarium (PEY).

**Table 1:** Analyzed species/varieties and their vouchers, locations, and ploidy levels.

Taxon	Ploidy level	Sample code	Locality	Voucher
*Chrysanthemum argyrophyllum* Ling	6x	HN	China: Henan, 34°25′N, 110°28′E	Rao GY 060718
*C. arisanense* Hayata	2x	TW	Taiwan: Mt. Arisan	Zhang ZX 081008
*C. boreale* (Makino) Makino	2x	KR	Korea: Chungcheongnam-do, Yeongi-gun36°29′N, 126°44′E	Jang CG 061019
*C. chanetii* H. Lévl.	2x	HLS	China: Ningxia, 38°44′N, 105°54′E	Rao GY 090313
*C. crassum* (Kitam.) Kitam.	10x	JP	Japan: Tokyo, Koishikawa Bot. Garden	Soejima A 1051
*C. dichrum* (C.Shih) H. Ohashi et Yonek.	4x	HB	China: Hebei, 37°35′N, 114°37′E	Rao et al. 050623
*C. glabriusculum* Hand.-Mazz.	2x	SC	China: Sichuan, 30°03′N, 101°59′E	Liang ZC 060913
*C. hypargyrum* Diels	4x	TB	China: Shaanxi, 33°58′N, 107°46′ N	Rao et al. 050717
*C. indicum* L.	4x	GZ	China: Guangzhou, 23°04′N; 113°19′E	Yang WH 010820
*C. indicum* L.	6x	JP	Japan: Tokyo, Koishikawa Bot. Garden	Soejima A 1046
*C. indicum* L.	4x	NJ	China: Nanjing, 32°03′ N, 118°37′E	Yang & Zhang 020609
*C. indicum* L.	4x	SNJ	China: Hubei, 30°58′ N, 110°01′E	Yang WH 030708
*C. indicum* var. *aromaticum* ( Q. H. Liu & S. F. Zhang ) J. M. Wang & Y. T. Wu	2x	SNJ	China: Hubei, 30°58′ N, 110°01′E	Yang WH 030709
*C. lavandulifolium* (Fish. ex Trautv.) Makino	2x	BJ	China: Beijing, 39°59′N, 116°18′ N	Liu PL 050428
*C. maximowiczii* Komar.	6x	XL	China: Inner Mongolia, 43°26′N, 116°08′E	Rao & Liang 060829
*C. mongolicum* Ling	2x	WL	China: Inner Mongolia, 40°40′N, 109°26′E	Rao & Liang 060907
*C. morifolium* Ramat. cv. *HSZ*	6x	BJ	China: Beijing, Beihai Park	Liu PL 070814
*C. morifolium* Ramat. cv. *ZY*	6x	BJ	China: Beijing, Beihai Park	Liu PL 070812
*C. naktongense* Nakai	2x	ZJ	China: Hebei, 41°01′N, 114°45′E	Rao & Liang 060724
*C. naktongense* Nakai	6x	NML	China: Hebei, 41°57′N, 117°43′E	Wang JW 050630
*C. nankingense* Hand.-Mazz.	2x	NJ	China: Nanjing, 32°03′N, 118°50′E	Yang & Zhang 020610
*C. occidentali-japonense* (Nakai) Kitam.	6x	JP	Japan: Tokyo, Koishikawa Bot. Garden	Soejima A 1047
*C. occidentali-japonense* var. *ashizuriense* Kitam.	6x	JP	Japan: Tokyo, Koishikawa Bot. Garden	Soejima A 1048
*C. okiense* Kitam.	4x	JP	Japan: Tokyo, Koishikawa Bot. Garden	Soejima A 1045
*C. oreastrum* Hance	4x	CB	China: Jilin, 41°57′N, 128°02′E	Rao & Liang 050828
*C. ornatum* Hemsley	8x	JP	Japan: Tokyo, Koishikawa Bot. Garden	Soejima A 1052
*C. potentilloides* Hand.-Mazz.	4x	SX	China: Shanxi, 35°17′ N, 111°54′E	Zhao HE 060828
*C. rhombifolium* (Ling & C. Shih) H.Ohashi & Yonek.	2x	CQ	China: Chongqing, 30°58′ N, 110°01′E	Rao et al. 060717
*C. sichotense* (Tzvelev) Vorosch.	4x	RS	Russia: Far East, Sikhote- Alih, 43°45′N, 135°13′E	Gorovoy & Balysev 050714
*C. sinuatum* Ledeb.	2x	RS	Russia: Altai, 50°22′ N, 87°37′E	Tribsch & Ehrendorfer 030726
*C. vestitum* (Hemsley) Stapf	6x	YC	China: Hubei, 30°36′N, 111°13′E	Rao et al. 060710
*C. yezoense* Maekawa	10x	JP	Japan: Ibaraki, 36°17′N, 140°16′E	Goto S 071103
*C. yoshinaganthum* Makino ex Kitam.	6x	JP	Japan: Tokyo, Koishikawa Bot. Garden	Soejima A 1050
*C. zawadskii* Herbich	6x	AR	China: Inner Mongolia, 47°17′N, 120°27′E	Rao & Liang 070723
*C. zawadskii* Herbich	4x	RS	Russia: Buryatia, 51°57′N, 107°04′E	Doudkin R 040723
*C. zawadskii* var. *acutilobum* Sealy	6x	KR	Korea: Kyoungsangbuk-do, 36°19′N, 128°25′E	Jang CG 060922
*C. zawadskii* var. *Alpinum* (Nakai) Kitam.	6x	KR	Korea: Kyoungsangbuk-do, 36°01′N, 128°41′E	Jang CG 060923
*C. zawadskii* var. *latilobum* (Maxim.) Kitamura	4x	KR	Korea: Kyoungsangbuk-do, Mt. Hwanghak-san36°22′N, 128°53′E	Jang CG 060924
*Ajania achilloides* (Turcz.) Poljakov ex Grubov	2x	IM	China: Inner Mongolia, 41°04′N, 107°04′ E	Zhao HE 060716
*A. fastigiata* (C. Winkl.) Poljakov	2x	XJ	China: Xinjiang, 43°51′N, 88°06′ E	Tan DY 070824
*A. przewalskii* Poljakov	2x	GS	China: Gansu, 34°55′N, 102°53′E	An YM 100731
*A. purpurea* C.Shih	4x	XZ	China: Xizang, 28°56′N, 87°31′E	Rao GY 100813
*Phaeostigma salicifolium* (Mattf.) Muld.	2x	QH	China: Qinghai, 35°47′ N, 102°40′E	Rao GY 100802
*P. variifolium* (Chang) Muld.	2x	SX	China: Shaanxi, 33°58′N, 107°46′ N	Rao et al. 050716
*Elachanthemum intricatum* Ling et Y. R. Ling	2x	IM	China: Inner Mongolia, 41°42′ N, 110°21′E	Rao & Liang 070906
*Kaschgaria brachanthemoides* (Winkl.) Poljakov	2x	XJ	China: Xinjiang, 43°51′N, 88°06′ E	Tan DY 070728
*Artemisia annua* L.	2x	BJ	China: Beijing, 40°01′N, 116°12′ E	Liu PL 060516
*Brachanthemum pulvinatum* (Hand.-Mazz.) Shih	2x	IM	China: Inner Mongolia, 39°21′N, 106°25′E	Rao & Liang 070911
*Stilpnolepis centiflora* (Maxim.) Krasch.		IM	China: Inner Mongolia, 38°03′ N, 107°37′E	Rao & Liang 070912

### Ploidy level examination

The DNA ploidy level of each individual sample was checked by means of flow cytometry using leaves dried in silica gel [36], [37]. To produce a standard control for the DNA ploidy level determination, traditional chromosome counting was conducted from root tips of a diploid sample of *Chrysanthemum chanetii*. Root tips were pretreated in a saturated water solution of alpha-bromonaphthalene for 2 h and then fixed in a solution of ethanol: acetic acid (3∶1) for one day. Chromosomes were stained with acetocarmine and counted under a microscope.

### DNA extraction, amplification, and sequencing

Total genomic DNA was isolated from silica-gel dried leaves using the 2× CTAB method [38] or using the Plant Genomic DNA Kit (TianGen Biotech, Beijing, China).

Seven chloroplast DNA loci, the intergenic spacers *psbA-trnH, trnC-ycf6, ycf6-psbM, trnY-rpoB, rpS4-trnT*, and *trnL-F*, and the *rpL16* intron were amplified and sequenced with universal primers [39], [40] (Table 2).

**Table 2:** Primers used for this study.

Locus	Primer name	Primer sequence	Reference or source
*psbA-trnH*	psbA	5′-GTTATGCATGAACGTAATGCTC-3′	[39]
	trnH^GUG^	5′-CGCGCATGGTGGATTCACAATCC-3′	[39]
*trnC-ycf6*	trnC^GCA^F	5′-CCAGTTCRAATCYGGGTG-3′	[39]
	ycf6R	5′-GCCCAAGCRAGACTTACTATATCCAT-3′	[39]
*ycf6-psbM*	ycf6F	5′-ATGGATATAGTAAGTCTYGCTTGGGC-3′	[39]
	psbMR	5′-ATGGAAGTAAATATTCTYGCATTTATTGCT-3′	[39]
*trnY-rpoB*	trnYretF	5′-CGAATTTACAGTCCGTCCCC-3′	[40]
	rpoBretR	5′-GGACATTGCGTCTATCCC-3′	[40]
*rpS4-trnT*	trnS^GGA^	5′-TTACCGAGGGTTCGAATCCCTC-3′	[39]
	trnT^UGU^R	5′-AGGTTAGAGCATCGCATTTG-3′	[39]
*trnL-F*	trnL5′UAA	5′-CGAAATCGGTAGACGCTACG-3′	[39]
	trnF^GAA^	5′-ATTTGAACTGGTGACACGAG-3′	[39]
*rpL16*	rpL16F71	5′-GCTATGCTTAGTGTGTGACTCGTTG-3′	[39]
	rpL16R1516	5′-CCCTTCATTCTTCCTCTATGTTG-3′	[39]
*CDS*	CDSII	5′-CTTSTMCWTGATGACATRATGGA-3′	[35]
	CDSIIa	5′-ATGRATGSCTCBCAYACACG-3′	this study
	CDSVb	5′-TGCATTCTTCAATATCTGTTCCMGT-3′	[35]
	CDSVa	5′-CRAAAGTGTCGAGATAATCATT-3′	[35]

**Figure 1:**
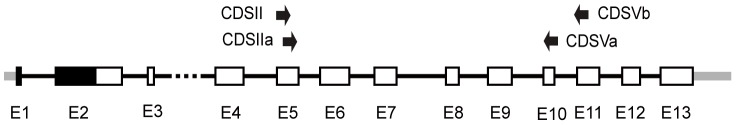
Structure of the *CDS* gene of *Chrysanthemum lavandulifolium* and the placement of primers. Gray, black, and white boxes represent 5′ and 3′ UTR, the encoding region of the predicted plastid targeting peptide and exons, respectively. Lines represent introns, and dots indicate the long intron. Arrows indicate the locations of the primers.

The nuclear chrysanthemyl diphosphate synthase (*CDS*) gene was amplified, cloned and sequenced. To verify the single-copy property of the *CDS* gene, its entire length (containing 13 exons and 12 introns) was sequenced from *Chrysanthemum lavandulifolium* and several species from other genera within Asteraceae. An N-terminal amino acid extension of 50 amino acids allowed the distinction of *CDS* from its paralogs (Liu et al. unpublished). For the present phylogenetic analysis, the region from exon 5 through 10 was sequenced using specific primers CDSII, CDSVb, CDSVa [35] and CDSIIa (designed by this study). The amplification was conducted first with the forward primer CDSII and the reverse CDSVb. This was followed by a nested PCR using the forward CDSIIa and the reverse CDSVa (Figure 1). Detailed information regarding the primers is given in Table 2.

The polymerase chain reaction was performed in a total volume of 20 μL containing 2 μL 10× buffer, 0.5 μM of each primer, 200 μM of each dNTP, 1 U of *exTaq* (TaKaRa, Shiga, Japan) or *Taq* polymerase (TianGen Biotech, Beijing, China), and 1 μL template genomic DNA. The amplification followed a program of 5 min at 95°C, 35 cycles of 1 min at 95°C, 1 min at 56°C, and 1 min at 72°C, and a final extension of 5 min at 72°C. The PCR products were excised from the 0.8% agarose gel and purified with a Gel Extraction Mini Kit (Watson Biotech, Shanghai, China). Direct sequencing was conducted for the chloroplast DNA fragments using the same primers as in the amplification. To sequence the nuclear *CDS* gene, the purified PCR products were ligated into a pGEM-T Easy Vector using a Promega Kit (Promega Corporation, Madison, WI, U.S.). Plasmids containing the right insertion were chosen for the sequencing reaction, and basically five clones were sequenced per individual. Cycle sequencing was conducted using the vector-specific universal T7 and SP6 primers, and the products were run on a 3730 automatic DNA sequencer. All sequences analyzed were deposited in GenBank (Accession Numbers JF939945-JF940511; JQ612544).

### Data analysis

Sequences were aligned with ClustalX version 1.81 [41] and then visually checked and modified with BioEdit version 7.0.1. Possible PCR-recombinant sequences [42]–[44] of the nuclear *CDS* were identified using method described by Russel *et al*. [45] and eliminated.

**Figure 2:**
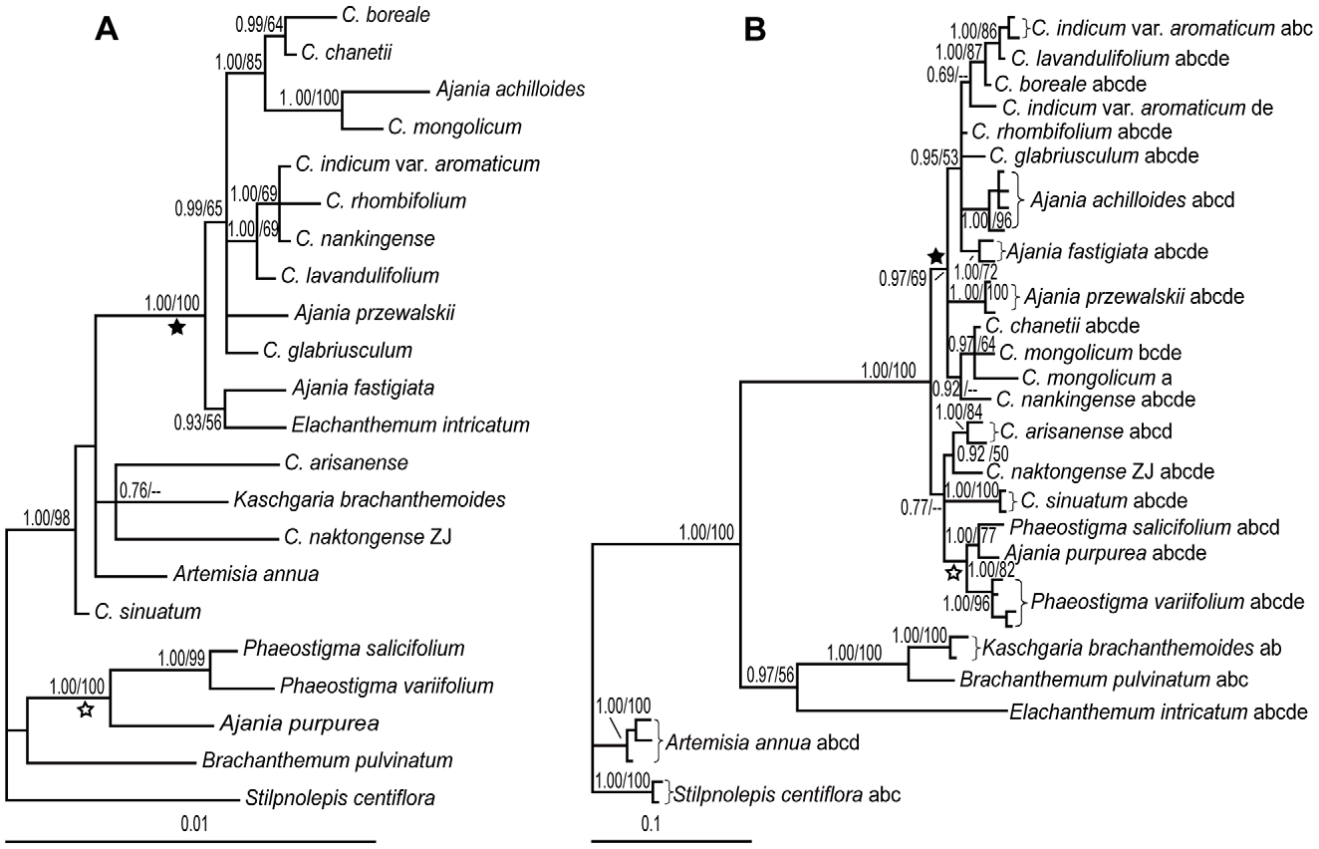
Bayesian 50% majority consensus trees of the diploid *Chrysanthemum* species and their generic allies. A: The tree of seven cpDNA loci combined. B: *CDS* exon gene tree. Posterior probabilities from the Bayesian inference (PP)/bootstrap percentages (BP) from the maximum parsimony analysis are shown next to the branches. “–” indicates where the BP value is <50% and the corresponding clade collapsed in the MP 50% bootstrap majority consensus tree. Main clades are indicated by ★/☆. Labels of terminal branches include species or variety name followed by population code (when available; in capital letters) and clone identities (from “a” to “e”; only for the CDS gene tree).

Because polyploids are common in *Chrysanthemum* and some of them are probably of hybrid origin, the resolution and topology of the phylogenetic trees may be interfered by the hybrid-polyploids [46]. To minimize this influence, we analyzed the present data in two steps: First, we focused on the circumscription and monophyly of *Chrysanthemum* and its relationships with the allied genera by excluding all the polyploid *Chrysanthemum* samples (Figures 2A and 2B). The monotypic genus *Stilpnolepis* at the relative basal position of phylogeny of the subtribe Artemisiinae [18], [19] was defined as the outgroup. Because the intron sequences of the *CDS* gene are hypervariable at the generic level and cannot be properly aligned across all the genera studied here, the *CDS* gene tree of the diploid *Chrysanthemum* and its allied genera (Figure 2B) was constructed using only their exon sequences. In the second step, we focused on the phylogeny of *Chrysanthemum* and all the *Chrysanthemum* species were included. *Stilpnolepis* was still used to root the cpDNA tree in this analysis. To obtain a better solution of relationships within *Chrysanthemum, CDS* intron sequences were also used. Because the *CDS* intron sequences of the *Chrysanthemum* species can only be aligned with those of *Ajania* and *Phaeostigma*, but not with any putative outgoups, samples of these two genera together with *Chrysanthemum* were analyzed and an unrooted tree was built on the the CDS exon+intron sequences.

Incongruence length difference (ILD) test [47] implemented in PAUP 4.0b10 [48] was used to assess the congruence of the data, between each pair of the seven chloroplast loci, between the exons and introns of *CDS*, and between the combined cpDNA and nuclear *CDS* data. When the test was conducted between chloroplast and nuclear DNA, only plant individuals lacking divergent nuclear haplotypes were used. Because *CDS* intron sequences only from the *Chrysanthemum, Ajania*, and *Phaeostigma* species could be aligned, the exons and introns from these samples were used to perform the ILD test. The test was performed with 1000 replicates, random taxon addition (10 replicates) and a maximum of 1000 trees saved per round. The results showed no significant incongruence between any pairs of the cpDNA loci (*p* ranges from 0.197 to 1.000) or between exons and introns of *CDS* (*P* = 0.884), but there was significant ionncgruence between the combined cpDNA and the nuclear *CDS* datasets (*P* = 0.001). The seven cpDNA loci were then combined for the analysis, and the exons and introns of CDS from *Chrysanthemum, Ajania*, and *Phaeostigma* were also combined for analysis. The chloroplast and nuclear data were analyzed separately.

Phylogenetic analyses were conducted using Bayesian inference (BI) and maximum parsimony (MP) methods. In all analyses, gaps were treated as missing data.

For the Bayesian analyses, the best-fit nucleotide substitution models were selected using Akaike information criterion in MODELTEST 3.06 [49]: TVM+I and K81uf+G for the cpDNA and *CDS* exon data sets (with the diploid *Chrysanthemum* species and all the allied genera), and GTR+I+G and K81uf+G for the cpDNA (covering all the samples) and *CDS* intron + exon data sets (covering all the *Chrysanthemum* species and *Ajania* and *Phaeostigma*), respectively. Bayesian inference (BI) was conducted in MRBAYES 3.1.2 [50]. Two independent MCMC runs, each with four chains (three hot, one cold) were run simultaneously starting from a random tree for 10 million generations with one tree sampled every 1000 generations. According to stationarity of lnL assessed using Tracer v. 1.4 [51], the first 10% sampled trees prior to stationarity were discarded as burn in for all analyses, and the remaining trees were used to construct the 50% majority-rule consensus tree.

Maximum parsimony analyses were performed with PAUP 4.0b10 [48]. Heuristic search was performed with random taxon addition with 1000 replicates, TBR branch swapping, and saving multrees (no more than 10 trees with the score no less than 10 were saved per replicate). The MP bootstrap analysis was conducted with 1000 replicates, simple sequence addition, TBR branch swapping, and multree option on.

### Examination of pollen

To examine the types of pollen variation among these genera, pollen grains of *Phaeostigma salicifolium, P. variifolium, Ajania purpurea, A. przewalskii, Chrysanthemum indicum*, and *C. naktongense* were collected from the voucher specimens used for the molecular analysis. Pollen grains were mounted on a metal stub, critical-point dried, and sputter-coated with gold. Observations and micrographs were performed under scanning electron microscopy (SEM, Hitachi S-4800) at 10 kV.

## Results

### Variations in ploidy level

The results of flow cytometry are listed in Table 1. Considerable variation in ploidy level was found within *Chrysanthemum*. Ploidy ranged from 2x to 10x. Infra-species variation was also observed. For instance, *C. indicum* was found to have 2x (SNJ), 4x (NJ, SNJ, and GZ), and 6x (JP) populations, and *C. zawadskii* was found to have 4x (RS) and 6x (AR) populations. In most cases, the diploid species appear to be endemic or narrowly distributed, and polyploids are usually widely distributed.

### Sequence characteristics

Seven chloroplast loci were each sequenced from 49 individuals of 43 taxa (Table 1). The combined cpDNA data set has 3971 characters, of which 227 are variable and 70 parsimony-informative. Of the seven plastid loci, the intergenic spacer *psbA-trnH* showed the highest percentage of informative characters as 4.90%, whereas *trnY-rpoB* the lowest as 0.84% (Table 3). Partial sequences of the nuclear *CDS* gene from its exon 5 through to exon 10 were sequenced. In total, 228 sequences were obtained from 49 individuals of 43 taxa (Table 3). These sequences fall into 150 haplotypes with a length variation from 879 bps (in *Artemisia annua*) to 1265 bps (in *Chrysanthemum sichotensis*). The aligned *CDS* exon data set covering all the studied taxa contains 429 nucleotide positions, of which 192 (44.76%) are variable and 184 (42.89%) parsimony informative (Table 3). The *CDS* exon + intron data set covering *Chrysanthemum, Ajania*, and *Phaeostigma* contains 1374 nucleotide positions, of which 418 (30.42%) are variable and 378 (27.51%) are parsimony informative (Table 3).

**Table 3:** Sequence characteristics of cpDNA and nuclear *CDS* gene.

Data sets	Number of taxa (total sequences)	Aligned length (range)	Number of variable sites (%)	Number of informative sites (%)
All samples				
*psbA-trnH*	43 (49)	429 (368–399)	36 (8.39%)	21 (4.90%)
*trnC-ycf6*	43 (49)	561 (493–549)	41 (7.31%)	13 (2.32%)
*ycf6-psbM*	43 (49)	615 (512–561)	53 (8.62%)	10 (1.63%)
*trnY-rpoB*	43 (49)	711 (627–685)	23 (3.23%)	6 (0.84%)
*rpS4-trnT*	43 (49)	370 (354–360)	19 (5.14%)	6 (1.62%)
*trnL-F*	43 (49)	374 (359–374)	18 (4.81%)	6 (1.60%)
*rpl16*	43 (49)	911 (843–873)	37 (4.06%)	8 (0.88%)
Combined cpDNA	43 (49)	3971 (3597–3699)	227 (5.72%)	70 (1.76%)
Nuclear *CDS* (exon)	43 (228)	429 (358–427)	192 (44.76%)	184 (42.89%)
*Chrysanthemum+Ajania+ Phaeostigma*				
*psbA-trnH*	38 (44)	416 (369–399)	32 (7.69%)	20 (4.81%)
*trnC-ycf6*	38 (44)	560 (493–549)	32 (5.71%)	13 (2.32%)
*ycf6-psbM*	38 (44)	609 (512–561)	33 (5.42%)	9 (1.48%)
*trnY-rpoB*	38 (44)	654 (627–636)	20 (3.06%)	5 (0.76%)
*rpS4-trnT*	38 (44)	369 (354–360)	10 (2.71%)	6 (1.63%)
*trnL-F*	38 (44)	374 (359–374)	14 (3.74%)	6 (1.60%)
*rpl16*	38 (44)	906 (845–873)	25 (2.76%)	7 (0.77%)
Combined cpDNA	38 (44)	3888 (3597–3691)	166 (4.27%)	66 (1.70%)
Nuclear *CDS* (exon+intron)	38 (211)	1374 (937–1265)	418 (30.42%)	378 (27.51%)

### Circumscription of *Chrysanthemum* and its relationships with the generic allies

To assess the circumscription of *Chrysanthemum*, only the diploid species of the genus and all its generic allies were included in the first step of analyses. The Bayesian inference and the maximum parsimony (MP) analyses generated almost the same topologies. The results of the MP analysis are only shown by bootstrap values mapped on the Bayesian trees (Figures 2A and 2B).

The close relationships of *Chrysanthemum* and *Ajania* are apparent in both the cpDNA and the *CDS* exon trees. Most *Chrysanthemum* and *Ajania* species formed a clade in the *CDS* exon tree (marked as ★ in Figure 2B), but *C. arisanense, C. naktongense* (population ZJ), *C. sinuatum* and *A. purpurea* were not included in this clade. In the cpDNA tree (Figure 2A), similar relationships were found except that *Elachanthemum* falls into the clade otherwise only of *Chrysanthemum*+*Ajania* (clade ★ in Figure 2A). *A. purpurea*, a tetraploid species, was grouped with two species of *Phaeostigma* in both trees (clades ☆ in Figure 2A & B). In the *CDS* exon tree, *Elachanthemum, Kaschagaria* and *Brachanthemum* formed a clade sister to that consisting of *Chrysanthemum, Ajania* and *Phaeostigma*, while the intergeneric relationships were not resolved by the cpDNA data, i.e., *Elachanthemum, Kaschagaria* and *Artemisia* were intermingled with the *Chrysanthemum*+*Ajania* group (Figure 2A). Therefore, *Elachanthemum, Kaschagaria*, and *Artemisia* were more close to *Chrysanthemum* than *Phaeostigma* and *Brachanthemum* do in the cpDNA tree, but the *CDS* gene tree suggests that *Phaeostigma* may be more closely related to *Chrysanthemum* and *Ajania* than all the other genera studied. The close relationship between *Phaeostigma* and the *Chrysanthemum+Ajania* is further supported by the fact that, among all the genera studied here, only *Phaeostigma* has *CDS* intron sequences that can be properly aligned with those of *Chrysanthemum* and *Ajania*.

### Phylogeny of *Chrysanthemum*

**Figure 3:**
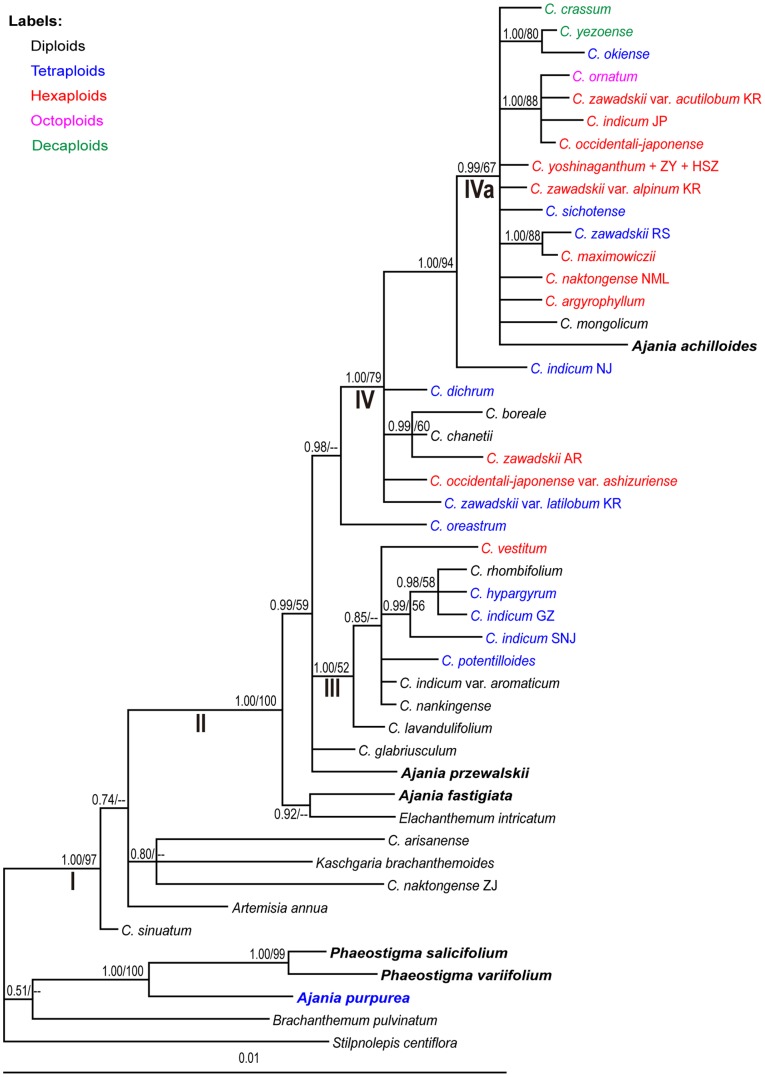
Bayesian 50% majority consensus tree of *Chrysanthemum* species and their generic allies. The tree is based on the sequences of seven cpDNA loci combined. Posterior probabilities from the Bayesian inference (PP)/bootstrap percentages (BP) from the maximum parsimony analysis are shown next to the branches. “–” indicates where the BP value is <50% and the corresponding clade collapsed in the MP 50% bootstrap majority consensus tree. Labels of terminal branches include species or variety name followed by population code (when available; in capital letters). Major clades are designated by roman letters. “ZY” and “HSZ” in the terminal branch “*C. yoshinaganthum* + ZY + HSZ” indicate to *C. morifolium* cv. ZY and *C. morifolium* cv. HSZ, respectively.

**Figure 4:**
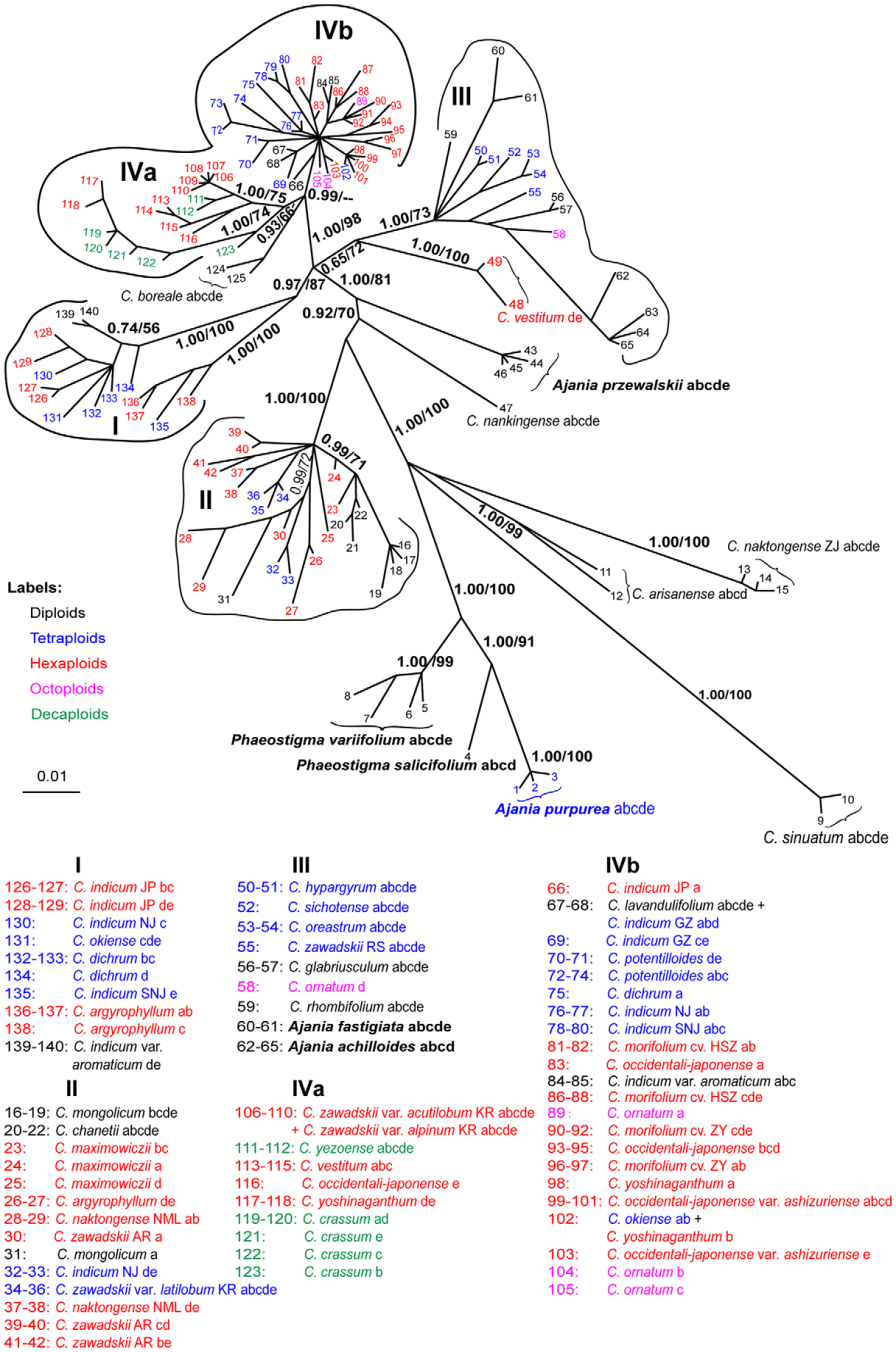
Bayesian 50% majority consensus tree of *Chrysanthemum*, *Ajania*, and *Phaeostigma*. The tree is based on the nuclear CDS exon+intron sequences. Posterior probabilities from the Bayesian inference (PP)/bootstrap percentages (BP) from the maximum parsimony analysis for the major clades are shown next to the nodes. “–” indicates where the BP value is <50% and the corresponding clade collapsed in the MP 50% bootstrap majority consensus tree. Labels of terminal branches contain: species or variety name followed by population code (when available; in capital letters), and then clone identities (from “a” to “e”).

Phylogenetic trees including polyploid *Chrysanthemum* species are shown in Figures 3 and 4 (an unrooted *CDS* intron+exon tree covering *Chrysanthemum+Ajania+Phaeostigma*).

In the cpDNA tree (Figure 3), most *Chrysanthemum* species fell into clade II. In this core group of *Chrysanthemum*, we recognize two major clades, III and IV. All the species in clade III are from China, and most of them have yellow ray florets and belong to the *C. indicum* group (*C. rhombifolium* and *C. vestitum* do not). In contrast, most of the species/varieties in clade IV have white-purple ray florets (*C. boreale, C. dichrum, C. indicum* (4x+6x), and *C. okiense* do not). In clade IV, subclade IVa includes all the species/varieties occurring in northern and northeastern China, Russia, the Korean Peninsula, and Japan.

Figure 4 is based on the *CDS* exon + intron sequences. As shown in Figures 2A, 2B, and 3, the three diploid species *C. sinuatum, C. naktongense* (ZJ), and *C. arisanense* show rather distant relationships with other species within *Chrysanthemum*. Again, we find that *Chrysanthemum* and *Ajania* cannot be separated. Four major clades, I, II, III, and IV can be recognized for the majority of the *Chrysanthemum* species and some of the *Ajania* species (Figure 4). Clade I broadly corresponds to the *C. indicum* group (characterized by small capitula with yellow ray florets) except for three haplotypes of *C. argyrophyllum*. Clade II contains species with white-purple ray florets. These belong to the *C. zawadskii* group except for two haplotypes of *C. indicum* (NJ) and two haplotypes of *C. argyrophyllum*. Clade III includes *Chrysanthemum* species with white-purple ray florets and two *Ajania* species. Except for the octoploid *C. ornatum*, Clade III contains diploid and tetraploid species homozygous at the *CDS* locus. Clade IV exhibits considerable variation with respect to *i)* ploidy levels from 2x to 4x, 6x, 8x, and 10x, *ii)* yellow and white-purple ray florets, and *iii)* a broad geographic distribution.

Many of the polyploid samples harbor divergent nuclear *CDS* gene haplotypes, which indicate their hybrid origins. For example, the tetraploid *C. indicum* (NJ) has its *CDS* haplotypes scattered in clades I, II, and IVb; and the hexaploid *C. indicum* (JP) harbors divergent *CDS* haplotypes found in I and IVb; the hexaploid *C. argyrophyllum* has haplotypes in clade I and II; and the hexaploid *C. vestitum* has two haplotypes close to clade III and three in clade IVa.

### Variations in pollen types

**Figure 5:**
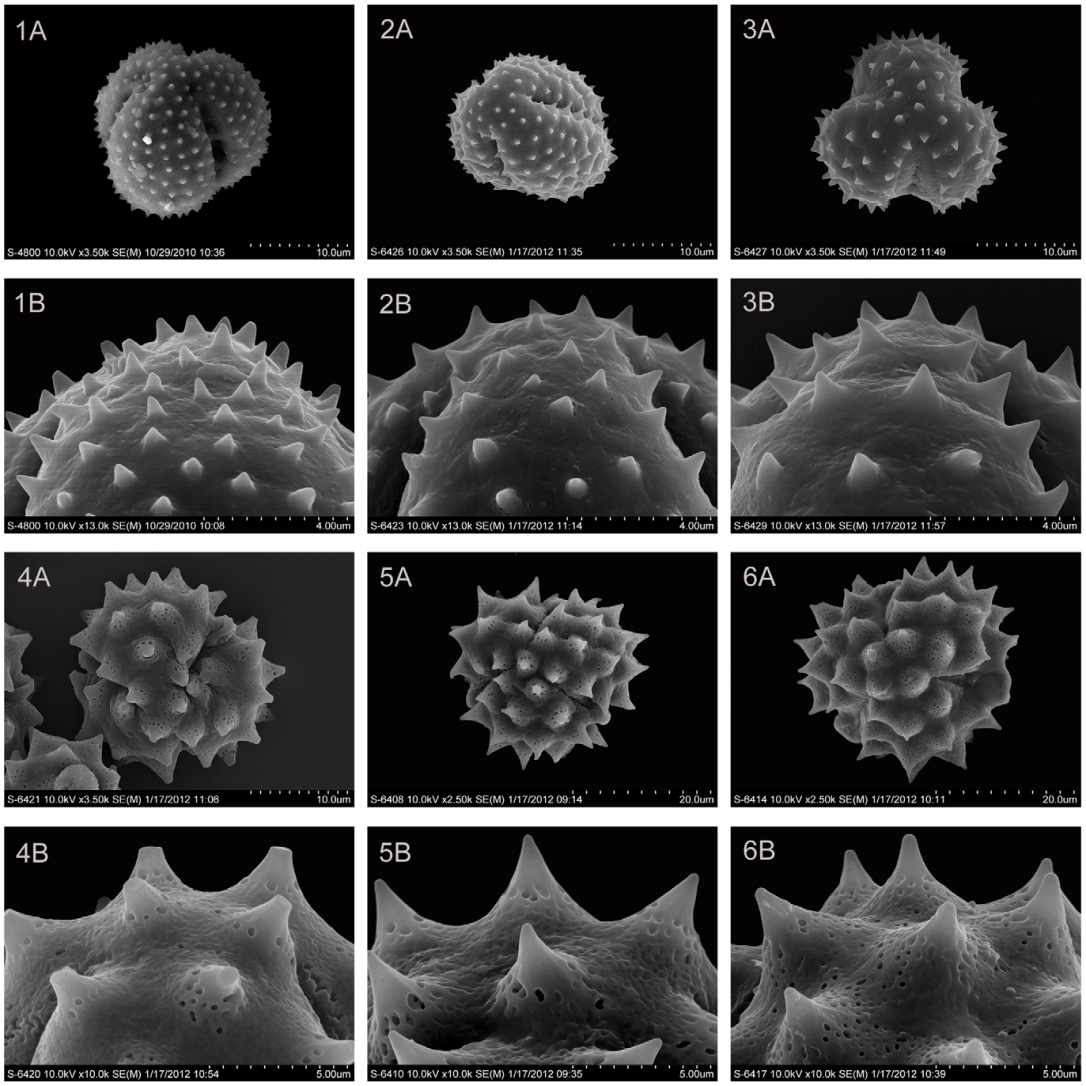
Scanning electron microscopy (SEM) micrographs of pollen grains. 1A and 1B: *Ajania purpurea*; 2A and 2B: *Phaeostigma salicifolium*; 3A and 3B: *P. variifolium*; 4A and 4B: *A. przewalskii*; 5A and 5B: *Chrysanthemum indicum*; 6A and 6B: *C. naktongense*.

The six species examined here have been found to have two types of pollen exine ornamentations. They are the *Anthemis*-type (echinate) for *Chrysanthemum indicum, C. naktongense*, and *Ajania przewalskii* and the *Artemisia*-type (microechinate) for *Phaeostigma salicifolium, P. variifolium*, and *A. purpurea* (Figure 5). For *P. variifolium* and *C. naktongense*, this is a first report of their pollen morphology. The close relationship between *Ajania purpurea* and *Phaeostigma*, reflected by pollen morphology, is in line with that indicated by DNA sequence data (Figures 2–4).

## Discussion

Previous studies based on nrITS, nrETS, and cpDNA data have provided insights into the phylogeny of the tribe Anthemideae [13], [14], [18]–[20]. They have also helped to resolve generic delimitation and circumscription of several groups within the subtribe Artemisiinae [13], [14], [18]–[20]. However, as Masuda *et al*. have pointed out, species of *Chrysanthemum* and its allies may have undergone rapid adaptive diversification so that they appear to be unresolved by using DNA markers (nrITS, nrETS and cp*trnL-F*) that have relatively low levels of divergence [14].

Our data show that, for *Chrysanthemum* and its allies, the chrysanthemyl diphosphate synthase (*CDS*) gene harbors rich information at both the generic (with its exon regions) and species (mainly with the highly variable intron regions) levels. It contains substantially more polymorphic sites than the combined cpDNA loci (Table 3) and the nuclear ribosomal ITS regions [13]. Together with the data from seven chloroplast DNA loci, which are of relatively rich in potentially informative characters (PICs) across the angiosperms and in Asteraceae [39], [40], the *CDS* gene sequences have helped us to develop a better understanding of the evolutionary history of *Chrysanthemum* and its allied genera.

### Monophyly, circumscription, and close relatives of *Chrysanthemum*

Both the cpDNA and nuclear gene trees (Figure 2A & 2B) failed to support *Chrysanthemum* as a monophyletic genus. The affinity of *Chrysanthemum* with *Ajania* indicated by the present study is in line with the results of several other studies based on the nrDNA ITS, ETS, and cpDNA data [13], [14], [18]–[20]. The genus *Ajania* was established by Poljakov based on its corymbose synflorescence of the disciform capitula [52]. He speculated that *Ajania* is a very old group derived from the same ancestors as *Artemisia* [52]. However, Tzvelev assumed a close affinity between *Ajania* and *Chrysanthemum* [53]. Masuda et al. suggested that *Chrysanthemum* (except for *C. sinuatum*) and *Ajania* were too close to be separated and that they should be treated within the genus *Chrysanthemum* sensu stricto [14]. According to our data, we agree with Masuda et al. that Ajania should be incorporated into *Chrysanthemum* despite their differences with respect to capitulum characters [14]. We also agree with Tzvelev that Ajania could have evolved from an ancestral taxon closely related to *Chrysanthemum* [53]. There are three types of capitula in the subtribe Artemisiinae: discoid, disciform, and radiate. According to recent studies, transcription factor *CYCLOIDEA*-like (*CYC*-like) genes play an important role in the morphogenesis of flower symmetry [54]–[56]. The disciform capitula of the *Ajania* species might be due to convergent genetic or epigenetic changes related to functions of the *CYC*-like genes.

With respect to the relationships between *Chrysanthemum*+*Ajania* and other allied genera, incongruence is evident between the cpDNA and nuclear *CDS* data: In the cpDNA tree, *Elachanthemum, Kaschgaria*, and *Artemisia* appear to be more close to *Chrysanthemum*+*Ajania* than *Phaeostigma* and *Brachanthemum* (Figure 2A). According the nuclear *CDS* data, however, *Phaeostigma* is more closely related to *Chrysanthemum*+*Ajania* than any other genera (Figure 2B). These conflicting relationships were also reported by other studies. The monophyly of the *Chrysanthemum* group ( = *Dendranthema*-group) including *Chrysanthemum, Ajania* and *Elachanthemum* was evident in several studies [13], [18]–[20]. However, the close affinity of *Phaeostigma* with *Chrysanthemum*+*Ajania*, especially with *Ajania*, was supported by the nuclear ribosomal ITS and ETS data and the capitulum traits [9], [14].

The incongruence between the plastid and nuclear gene trees can be attributed to hybridization, incomplete lineage sorting, and different evolutionary rates and history of plastid and nuclear genomes or DNA regions, [57], [58]. These factors make it difficult to interpret gene trees with any certainty. The major problems are the differentiation of speciation genes, discrepancy between phenotypic traits and the randomly selected DNA regions used for the phylogenetic survey, different re-combinations of parental genomes, and different patterns of lineage sorting during subsequent species radiations.

Retention of ancestral polymorphisms, or incomplete lineage sorting, is common in recently diverged species [57]. Relevant problems have been evident in many recently radiated groups of the Asteraceae, such as *Tragopogon* and *Achillea* [59]–[61]. In *Achillea*, by comparing the inferred species tree and the nuclear gene trees, Guo *et al*. demonstrated that lack of sorting of ancestral alleles exists not only among closely related species but extends to the taxa with relatively distant relationships [61]. In the present study, the incongruence between the plastid and nuclear *CDS* trees already on the 2x-level in *Chrysanthemum* and its generic allies mainly stem from lack of lineage sorting and/or hybridization. Hybridization within *Chrysanthemum* will be discuss in a later chapter. Here, we interpret the inter-generic relationships given the available molecular and morphological data.

Considering that *i*) plastid DNA variation is often too low to support the inference of species relationships, *ii*) *Phaeostigma* is the only genus among the allied genera that can be compared to *Chrysanthemum* + *Ajania* in terms of *CDS* intron sequences, and *iii*) *Phaeostigma* and *Ajania* both have disciform-heterogamous capitula, we conclude that *Phaeostigma* is more closely relative of the *Chrysanthemum* + *Ajania* group than *Elachanthemum*.

*Phaeostigma* is a small genus consisting of only three species. Muldashev separated it from *Ajania* on the basis of its brownish style-branches, erect corolla lobes and pollen characters [62]. According to Muldashev, *Phaeostigma* is closely related to the ancestors of *Ajania* and distantly related to *Artemisia* even though it has the same pollen morphology as the latter [62]. There are two types of pollen exine ornamentations in the subtribe Artemisiinae [20]–[22]. *Phaeostigma* has *Artemisia*-type pollen, and *Ajania* has *Anthemis*-type pollen [9], [21]. Bremer and Humphries proposed that *Phaeostigma* might be a grade between *Ajania* and *Artemisia* [9]. They also suggested that circumscribing *Phaeostigma* requires more extensive study on greater numbers of *Ajania* species, especially those with habits similar to those of *Phaeostigma*. Our field observations showed that *Phaeostigma* plants have larger leaves and grow in more humid habitats than most *Ajania* species.

The distinction between *Phaeostigma* and *Ajania* seems to be difficult due to the placement of *Ajania purpurea* in the clade of *Phaeostigma*, which is placed with confidence by both gene trees (Figures 2A & 2B). Pollen traits show that *A. purpurea* shares with *Phaeostigma* the *Artemisa*-type, unlike the *Anthemis*-type of *A. przewalskii* (Figure 5) and other *Ajania* species [20]–[22]. *A. purpurea* is a small, tetraploid perennial herb. It grows in rock fissures on mountain slopes, gravel mounds, and alpine meadows at elevations of 4800–5300 m in Xizang (Tibet). In addition to pollen traits, it shares many other morphological features with *P. variifolium*, as indicated by the results of our field observations. For instance, their disciform capitula have urceolate involucres consisting of 4 rows of involucral bracts with dark brown margins. The disc florets have brownish style-branches and erect corolla lobes. Taking both the molecular [Figure 2] and morphological data into consideration, we regard the tetraploid *A. purpurea* as a member of *Phaeostigma*, which has been adapted to the alpine environment probably via polyploidization.

With respect to other three genera, *Elachanthemum, Kaschgaria*, and *Brachanthemum*, cpDNA data show them to be quite divergent (Figure 2A), but nuclear *CDS* exon data place them in a clade sister to the *Chrysanthemum+Ajania+Phaeostigma* group (Figure 2B). Given the pattern of molecular and morphological variations, particularly pollen morphology, the relationships among these genera are in agreement with Tzvelev's hypothesis [53], i.e., the radiate-heterogamous and disciform-heterogamous taxa may have evolved from the same ‘dendranthemoid’ ancestor.

### Main lines of phylogeny of *Chrysanthemum*

Both the cpDNA and nuclear *CDS* gene data suggest that *C. sinuatum, C. arisanense*, and *C. naktongense* (ZJ population) are distantly related to the main lineage of *Chrysanthemum* sensu stricto ( = *Chrysanthemum+Ajania*) (Figures 2–4). *C. sinuatum*, and *C. arisanense* are diploid species (Table 1 & reference [12]), and *C. naktongense* has various ploidy levels (2x to 4x and 6x) (Table 1). The population ZJ of *C. naktongense* that is clustered with *C. arisanense* and close to *C. sinuatum* is diploid. These three species are morphologically distinct and geographically separated. *C. sinuatum* is a narrowly distributed species only in Altai, Russia [12]. It has pink ray florets and leaves with 2–5 pinnatipartite or pinnatisect. While, *C. arisanense* is endemic to Taiwan and has yellow ray florets and leaves with 1–2 pinnatipartite [8]. *C. naktongense* is quite different from the former two. It is polymorphic not only in ploidy but also in morphology, and therefore, there was much controversy over its species identity [8], [9], [63]. The present data also show marked molecular differences for two accessions of *C. naktongense* (ZJ and NML). In our opinion, this is not a good species, and it needs multidisciplinary data from multiple populations to resolve this species problem.

*Chrysanthemum* has the species diversity center in China. According to the geographic distribution and morphology, the Chinese *Chrysanthemum* species can be divided into two species groups, the *C. indicum* group (clade III in Figure 3 and mainly clade I in Figure 4) and the *C. zawadskii* group (clade IVa in Figure 3 and mainly clade II in Figure 4). The *C. indicum* group is distributed in southern China. It is mainly represented by *C. indicum* with creeping stems and small capitula with yellow ray florets. In contrast, the *C. zawadskii* group occurs in northern China, predominated by *C. zawadskii* with erect stems and large capitula with white-purple ray florets. At the diploid level, *C. lavandulifolium* and *C. nankingense* belong to the *C. indicum* group, and *C. mongolicum* and *C. chanetii* are of the *C. zawadskii* group.

In the *CDS* gene tree (Figure 4), more geographic patterns can be recognized: Clade III is composed of narrowly distributed *Chrysanthemum* species in alpine or isolated mountains except two *Ajania* species. Within clade IV, molecular variation shows geographic structure. For example, the group IVa contains species/varieties mostly from Japan and Korea except three haplotypes of the hexaploid *C. vestitum*, and IVb only consists of those from China and Japan. Two hexaploid chrysanthemum cultivars, *C. morifolium* cv. HSZ and *C. morifolium* cv. ZY, are both nested in the clade IVb, indicating their origins probably from progenitors of this lineage.

Despite some incongruence, data from the plastid and nuclear DNA all show the geographic structure of the infrageneric diversification within *Chrysanthemum*. There are also many endemic species in *Chrysanthemum*, and most of them, such as *C. hypargyrum, C. rhombifolium, C. yoshinaganthum*, and *C. yezoense*, only occur in a specific habitat [1], [4], [8], [9]. All these findings suggest that the geographic and ecological speciation plays an important role in the evolution of the genus *Chrysanthemum*.

### Hybridization and polyploidy in the evolution of *Chrysanthemum*

Previous studies have demonstrated that polyploidy and hybridization are common in *Chrysanthemum* [1]–[5]. Both the nuclear and the cpDNA data presented here give some clues to the participation of various diploids in the origins of the polyploid taxa. For example, the diploids *C. lavandulifolium* and *C. nankingense* may have been involved in the origin of the polyploids of the *C. indicum* group (Figures 3 and 4). This conclusion is supported by previous studies [2], [24], [25], [64], [65]. Similarly, the diploids *C. mongolicum* and *C. chanetti* may have been involved in the origin of the polyploids of the *C. zawadskii* group (Figures 3 and 4). Within each group, species are very similar morphologically, but the diploid can be distinguished from those of polyploids by their leaf shape and relatively small capitula [2], [5].

Polyploidy via interspecific hybridization (allopolyploidy) is a major mechanism of speciation in plants [66], [67]. This mechanism can be involved in the speciation of some polyploid species in *Chrysanthemum*, e.g., *C. argyrophyllum*. This is a perennial hexaploid species occurring in the rock crevices of mountain peaks in NW Henan and SE Shaanxi Provinces. In the cpDNA tree (Figure 3), the haplotype of *C. argyrophyllum* is resided in the clade IVa that includes the diploid *C. mongolicum*. In the nuclear *CDS* gene tree (Figure 4), three haplotypes of *C. argyrophyllum* are clustered with the tetraploid *C. indicum* (SNJ) in clade I, and another two haplotypes are nested in clade II close to the diploid *C. mongolicum*. Morphologically, *C. argyrophyllum* is more close to *C. mongolicum* than *C. indicum* by sharing more common morphological characters, e.g., leaf-like involucral bracts and white-purple ray florets [8]. Thus, all the evidence from cpDNA, nuclear *CDS* and morphology support that *C. argyrophyllum* is an hexaploid species with hybrid origin, and diploid *C. mongolicum* may be its maternal progenitor.

The current study produced little evidence of autopolyploidy. This is consistent with earlier studies [68]. The 4x population GZ of *C. indicum* may be autopolyploid, because *C. indicum* (GZ) is nested in the clade III of cpDNA tree including the diploid *C. lavandulifolium* and the *CDS* haplotypes of *C. indicum* (GZ) only form a clade with that of *C. lavandulifolium*. In addition, the data from RAPD and ISSR markers showed that only the *C. lavandulifolium*-specific bands in those of three putative diploid progenitors can be found in the tetraploid *C. indicum* (GZ) [2]. All evidence was consistent with the possibility that *C. indicum* (GZ) might have an autopolyploid origin.

Considering all the evidence from the present molecular data and previous studies [1], [2], we conclude that geographic and ecological conditions as well as hybridization and polyploidy have played important roles in the divergence and speciation of the genus *Chrysanthemum*.
